# Empowering Pharmacists in Heartburn Management: Practical Insights for OTC Treatment and Self-Care

**DOI:** 10.3390/pharmacy13050124

**Published:** 2025-09-02

**Authors:** Mary Barna Bridgeman, Ashok Hospattankar, Kamran Siddiqui, Nardine Nakhla

**Affiliations:** 1 Ernest Mario School of Pharmacy, Rutgers, The State University of New Jersey, Piscataway, NJ 08901-8554, USA; 2 US Medical Affairs, Haleon, Warren, NJ 07059, USA; ashok.x.hospattankar@haleon.com (A.H.); kamran.h.siddiqui@haleon.com (K.S.); 3 School of Pharmacy, University of Waterloo, Waterloo, ON N2L 3G1, Canada; nnakhla@uwaterloo.ca

**Keywords:** heartburn, acid reflux, pharmacist, over-the-counter medications, patient safety, therapy optimization

## Abstract

Heartburn is a prevalent and frequently self-managed condition, with a myriad of over-the-counter (OTC) treatment options available for self-care. The potential for misinterpretation of drug labels and improper OTC medication selection may result in inadequate treatment, potential drug interactions, as well as medication overuse, misuse, or delay in seeking treatment for a more serious health condition. As highly accessible healthcare professionals, pharmacists play a crucial role in validating self-diagnoses, in guiding appropriate OTC medication selection and use, and in educating patients on both pharmacologic and non-pharmacologic management strategies for heartburn. It is essential for pharmacists to remain informed about the latest developments in disease management and treatment options. This narrative review provides an updated perspective on the epidemiology, risk factors, pathophysiology, and clinical manifestations associated with heartburn while underscoring the expanding role of pharmacists in patient care. This review includes a structured assessment framework and clinical management algorithm designed to enhance pharmacists’ ability to identify red flag symptoms, optimize OTC medication use, and facilitate timely referrals when necessary. By incorporating evidence-based guidance with patient-centered counseling, pharmacists can enhance treatment outcomes, optimize, medication use, promote adherence, and ensure safer self-care practices. As self-medication trends and the role of pharmacists evolves, this review offers a comprehensive resource to equip pharmacists with the latest knowledge and practical tools for optimizing heartburn management and promoting patient safety.

## 1. Introduction

The American Gastroenterological Association (AGA) defines heartburn as a burning sensation beneath the sternum radiating from the upper abdomen to the neck, and recognizes that this condition represents a hallmark symptom of gastroesophageal reflux disease (GERD) [1,2]. Contrary to popular belief, heartburn is not caused by excessive stomach acid production but occurs when gastric acid flows back into the esophagus. This backflow of acid is due to its misplacement in the digestive tract rather than gastric acid overproduction [3]. Heartburn synonyms, include “acid indigestion”, “acid regurgitation”, “sour stomach”, “hyperacidity”, and “acidity” [4].

In the United States (U.S.), heartburn is highly prevalent, with significant implications for public health [5,6]. Although generally non-fatal, chronic or poorly managed heartburn can result in serious complications including esophagitis, ulcers, peptic strictures, and Barrett’s esophagus, a premalignant condition that increases the risk of esophageal adenocarcinoma [7]. Beyond these physical morbidities, heartburn significantly impacts sleep, mental well-being, work productivity, social functioning, and dietary behaviors, thereby compromising overall quality of life. These disruptions contribute to substantial healthcare expenses and financial burden, which have been previously described [8,9,10]. The pathophysiology of heartburn is multifactorial and often exacerbated by modifiable risk factors, including obesity, high-fat diets, smoking, alcohol consumption, stress, and the use of specific medications [11].

A growing concern in the management of heartburn is the role of widely prescribed medications, particularly those used for chronic conditions, in triggering or exacerbating symptoms. Notably, glucagon-like peptide-1 receptor agonists (GLP-1RAs), commonly used for the treatment of type 2 diabetes mellitus (T2DM) and obesity, may be associated with gastrointestinal side effects including nausea, delayed gastric emptying, and heartburn, [12,13,14,15] stemming from their impact on delaying gastric motility and increasing gastric acid exposure in the esophagus [12]. In addition, numerous medication classes, including calcium channel blockers, bisphosphonates, non-steroidal anti-inflammatory drugs (NSAIDs), antiplatelet medications such as aspirin, anticholinergics, selective serotonin reuptake inhibitors (SSRIs), and serotonin/norepinephrine reuptake inhibitors (SNRIs) among others, can contribute to heartburn via various mechanisms [16,17,18,19,20]. Given the widespread use of these medications, pharmacists play a critical role in identifying and managing drug-induced gastrointestinal symptoms in patients presenting with heartburn, to ensure safe and effective self-care, including triaging patients to their prescriber, when appropriate.

With the increasing prevalence of heartburn across various age groups, many individuals turn to self-management strategies, including the use of over-the-counter (OTC) medications. OTC treatment options, including antacids, histamine-2-receptor antagonists (H2RAs), and proton pump inhibitors (PPIs), are commonly sought medications used to alleviate heartburn symptoms [1,3]. In the U.S., the demand for these medications is rising, as evidenced by the growth in antacid sales from $1.856 billion in 2019 to $2.025 billion in 2023, with similar trends observed for H2RAs and PPIs [21]. This increase in sales underscores the widespread occurrence of heartburn and the increasing reliance on OTC treatments for symptom relief.

Despite the availability of effective OTC treatments, patients may struggle with selecting the most suitable options for managing their heartburn symptoms [22]. A 2003 study conducted by the National Council on Patient Information and Education (NCPIE) found that 66% of Americans (n = 1009) reported difficulty in choosing an appropriate OTC medication due to the wide range of competing products available [23]. This confusion can lead to the inappropriate selection of an OTC medication, incorrect dosing, drug interactions, and delays in recognizing conditions that require medical evaluation. Misinterpretation of OTC labeling and confusion caused by combination products and brand extensions further complicate the decision-making process in self-managing heartburn.

Given the increasing reliance on self-medication, pharmacists are uniquely positioned to play a key role in ensuring the safe and effective use of OTC heartburn medications. As accessible healthcare professionals, pharmacists can assess the clinical presentation of heartburn (e.g., heartburn frequency, nature, duration of symptoms), confirm self-diagnoses, screen for contraindications, and provide evidence-based recommendations for therapy selection [6,24]. Pharmacists’ expertise extends beyond pharmacotherapy to include patient education, lifestyle counseling, and the early identification of alarm symptoms that necessitate medical referral for further diagnostic evaluation. This comprehensive, patient-centered approach enhances treatment efficacy, promotes long-term symptom control, and minimizes the risks associated with inappropriate self-medication [25,26].

This narrative review synthesizes contemporary evidence on the epidemiology, pathophysiology, and risk factors associated with heartburn, with a focus on the expanding role of pharmacists in guiding OTC heartburn management. By presenting a structured framework for clinical assessment and therapeutic counseling, the review aims to empower pharmacists to optimize self-care and enhance their impact on public health.

## 2. Methodology

This narrative literature review aimed to synthesize contemporary evidence related to the epidemiology, pathophysiology, clinical presentation, OTC pharmacologic strategies, and pharmacist-led interventions in the self-care management of heartburn, particularly in the context of polypharmacy. To ensure comprehensive coverage, targeted searches were conducted across PubMed, Embase, Medline, and Google Scholar using free-text keywords combined with Boolean operators and truncations. Keywords included “Heartburn”, “acid indigestion”, “acid regurgitation”, “sour stomach”, “hyperacidity”, and “acidity”, “Gastroesophageal Reflux Disease”, “GERD”, “Pharmacist Intervention”, “OTC Management”, “Antacids”, “H2 Receptor Antagonists”, “Proton Pump Inhibitors”, “Polypharmacy”, “Self-Care”, “Guidelines”, and “Patient Education”. A wide range of literature was considered, including randomized controlled trials, observational studies, clinical guidelines, product labeling, U.S. Food and Drug Administration (FDA) guidance documents, expert commentaries, and relevant grey literature. Citation tracking was employed to identify additional pertinent sources beyond the initial search results.

Insights were extracted through full-text review and thematic analysis, focusing on domains such as symptom classification, OTC treatment algorithms, drug interactions, patient counseling strategies, and pharmacist-driven care models. Images and flowcharts were created using Adobe Photoshop (version 26.6) and Adobe Illustrator (version 29.5), with contributions from the lead and co-authoring pharmacists, to illustrate therapeutic pathways and decision-making frameworks relevant to pharmacy practice. To ensure clinical relevance and alignment with current standards, guidelines from the American College of Gastroenterology (ACG), FDA OTC Drug Monographs, and the World Gastroenterology Organization (WGO) were consulted. Given the broad scope and conceptual nature of the review’s objectives, a traditional systematic meta-analysis was deemed unsuitable; instead, a narrative review approach was adopted to allow for integrative and practice-oriented synthesis. The review was planned, conducted, and reported in accordance with the Scale for the Assessment of Narrative Review Articles (SANRA). The completed checklist is available as supplemental materials (Supplementary Table S1) [27].

## 3. Results and Discussion

### 3.1. Epidemiology and Risk Factors of Heartburn

Heartburn affects an estimated 10–20% of the U.S. population daily [28,29]. According to the WGO Handbook on Heartburn, the incidence of heartburn was reported 17.8% in 2015 in the U.S. [30]. Heartburn is considered quite common in North America, whereas it is moderately prevalent in South America [31]. A 2018 nationwide survey of 71,000 participants revealed that 30.9% experienced heartburn or acid reflux symptoms [32]. However, there is a lack of recent data specifically on the incidence of heartburn, as most studies focus on GERD, which includes heartburn as hallmark symptom.

Although heartburn is traditionally associated with affecting middle-aged and older adults, it is an increasing concern among younger adults due to various risk factors, which may [33] include obesity, high-fat diet, smoking, alcohol consumption, pregnancy, certain medications, stress, gender, and age [5,33,34]. Table 1 and Table 2 provides a summary of the key risk factors reported in clinical studies (Table 1, medication-related risk factors for heartburn and Table 2, lifestyle-related risk factors for heartburn).

Notably, individual triggers for heartburn can vary significantly. For instance, some individuals report coffee as a trigger, while others report no symptoms with its consumption [35]. Moreover, many individuals find it challenging to accurately identify the specific causes of their symptoms. To mitigate these uncertainties, research is currently being conducted to elucidate the relationship between molecular receptors and their corresponding triggers. Based on emerging evidence, healthcare professionals may suggest general lifestyle modifications, including dietary changes, weight reduction, and alterations in body positioning to manage symptoms [1,36].

**Table 1:** Risk factors for heartburn. Medication-Related Risk Factors for Heartburn.

Drug Class and Medications	Mechanism of Heartburn Adverse Effects	Description of Incidence	Clinical Implications	References
NSAIDs (e.g., diclofenac, aspirin, ibuprofen)	Direct topical mucosal irritancy + systemic prostaglandin inhibition → acid-mediated injury adjacent to lower esophageal sphincter (LES)	Heartburn reported to occur in up to 1/3 of patients utilizing NSAIDs	Analgesia, anti-inflammation	[37,38,39]
Antibiotics (e.g., tetracyclines including doxycycline, clindamycin, amoxicillin, metronidazole, fluoroquinolones)	Low pH may irritate esophagus, resulting in dyspepsia and heartburn symptoms; drug-induced esophagitis	Incidence varies with medication; tetracyclines implicated in nearly 70% of all reported cases of drug-induced esophageal ulceration	Acne, atypical respiratory & zoonotic infections	[40,41]
Bisphosphonates (e.g., alendronate, risedronate, ibandronate)	Topical irritant to the esophagus, may induce esophageal Inflammation and damage; drug-induced esophagitis	Regurgitation and heartburn are reported to occur in more than 60% of patients.	Osteoporosis, Paget’s disease	[42]
Asthma treatment (e.g., theophylline, corticosteroids)	Theophylline use causes relaxation of LES and stimulation of gastric acid secretion, whereas systemic corticosteroids may increase stomach acid, affect the LES tone, and cause gastric irritation	Heartburn was reported in 73% (11 of 15) of subjects receiving theophylline, compared to 1 of 9 in the placebo group.	Bronchodilator for asthma	[43,44]
GLP1-RAs (e.g., liraglutide, semaglutide, exentide, dulaglutide)	Delaying gastric emptying, increasing intragastric pressure, and promoting transient relaxation of the LES	Dyspepsia reported to occur in 4–10% of adults receiving liraglutide, in 3–9% of adults receiving subcutaneous semaglutide, in 3–7% of adults receiving exenatide, and in 4–6% of adults receiving dulaglutide	T2DM and weight management	[45,46,47,48,49,50]

**Table 2:** Risk factors for heartburn. Lifestyle-Related Risk Factors for Heartburn.

Factor	Description	References
Obesity	Overall prevalence of gastroesophageal reflux was 62.4% in 2019 among obese patients in the U.S. (n = 242) Obesity was associated with an increased esophageal symptom burden and higher acid/bolus reflux level	[51,52]
High-fat diet	Possible reflux-triggering factor due to its ability to lower esophageal sphincter pressure and increasing acid exposure time	[53]
Tobacco smoking	Associated with an increased risk of gastroesophageal reflux symptoms (GORS), including heartburn. The adjusted odds ratio (OR) was 1.71 (95% CI 1.36–2.14) for any GORS and 1.80 (95% CI 1.10–2.93) for frequent GORS (n = 7620)	[54]
Alcohol consumption	Esophageal irritant and trigger of reflux and symptoms of GERD	[55]
Pregnancy	Estimated to affect 22% to 52% of women during first trimester of pregnancy, with prevalence increasing as gestational age progressed	[56]
Stress	High stress levels (reported by 62% of individuals experiencing stress at home or workplace) are reported as the risk factors for developing gastric symptoms, including heartburn (n = 100)	[57]
Gender	Risk of frequent GORS was found to be lower among boys as compared to girls (OR 0.61; 95% CI 0.46–0.79) (n = 7620; aged 13–19 years) Gender disparities exist in GERD complications, with 58.4% of the 27.2 million affected individuals in the U.S. being female.	[54]
Age	Individuals with >50 years of age were at an increased risk of developing GERD symptoms, including heartburn (n = 163,018)	[58]
Hiatal hernia	Hiatal hernia was found to be the most predominant associated factor for GERD symptoms, including heartburn	[59]

### 3.2. Pathophysiology and Symptoms of Heartburn

Heartburn occurs due to the retrograde flow of gastric acid from the stomach into the esophagus, a condition known as gastroesophageal reflux. Normally, this backflow is prevented by the lower esophageal sphincter (LES), a specialized muscular ring at the junction between the esophagus and stomach. The LES maintains a high-pressure zone that acts as a barrier, prevents gastric contents from entering the esophagus. However, when the LES relaxes inappropriately or weakens, acidic gastric contents can reflux into the esophagus, leading to the characteristic burning sensation of heartburn [3,36].

The exact molecular mechanism underlying the sensation of heartburn remains unclear; however, heartburn symptoms are thought to arise from the complex interplay of chemical, mechanical, and neural factors that activate sensory pathways in the esophagus. Recent studies have identified various acid-sensitive ion channels such as transient receptor potential (TRP) channels, acid-sensing ion channels (ASICs), ionotropic purinoreceptor channels (P2X), and acid-sensitive tandem-pore potassium channels (K2P/TASK), as well as chemosensory receptors, expressed within gastrointestinal sensory neurons. These ion channels may contribute to the detection and transmission of noxious stimuli. Additionally, neuropeptide-associated pathways, including those involving substance P and calcitonin gene-related peptide (CGRP), are thought to play a key role in amplifying sensory signaling and promoting inflammation, further sensitizing the esophageal mucosa [60].

**Figure 1:**
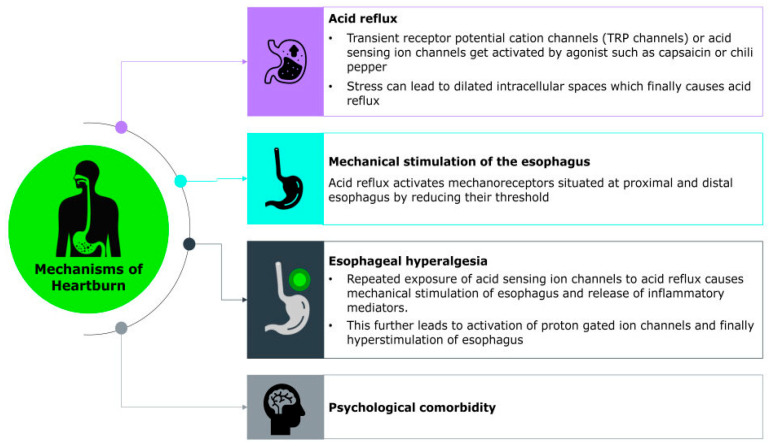
Molecular Mechanism of Heartburn.

A major trigger for this cascade is the reflux of gastric contents, including acid, bile, pepsin, and even weakly acidic substances, which directly stimulate chemoreceptors in the esophageal lining. Among these, the transient receptor potential vanilloid 1 (TRPV1) channels, also known as the capsaicin receptor, represent a promising candidate for mediating heartburn sensation during acid exposure by depolarizing sensory neurons and transmitting signals on perception of heartburn to the central nervous system [60,61] (Figure 1). Their clinical relevance is underscored by findings that TRPV1-positive cell density significantly decreases following radiofrequency ablation in patients with reflux hypersensitivity, coinciding with marked symptom relief [62]. The study also highlights the role of PAR-2 and CGRP-positive nerve fibers in reflux hypersensitivity, both contributing to heightened esophageal pain perception independent of mucosal damage. Importantly, psychological factors such as stress and anxiety can modulate this sensory experience. These conditions may lower pain thresholds and enhance central processing of esophageal stimuli, thereby intensifying symptom perception even in the absence of significant mucosal injury [62].

Furthermore, a 2025 pilot study by Mokrowiecka et al. reported elevated expression of purinergic receptors specifically P2X2, P2X3, and P2Y2 in the esophageal mucosa of individuals with nonerosive reflux disease (NERD). This upregulation correlates with symptom severity such as heartburn and is associated with dilated intercellular spaces, a hallmark feature of esophageal hypersensitivity [63].

As per the WGO guidelines, the primary symptom of heartburn is a burning chest pain, typically occurring after meals or at night. These symptoms are often exacerbated when lying down or bending over [31]. Patients typically describe heartburn as a burning sensation originating from the stomach or lower chest and rising toward the neck. This discomfort may also be felt as a warm or acidic sensation in the epigastrium or substernal areas, or as a feeling of fullness or discomfort in the epigastrium [64,65,66]. Additional symptoms may include bloating, stomach pain, chronic cough, sore throat, food regurgitation, and a bitter or acidic taste in the mouth. Heartburn is usually accompanied by regurgitation, where stomach contents travel back up into the throat or mouth. Patients frequently report that this symptom can disrupt sleep (nocturnal heartburn), contributing to a cycle of anxiety and further gastrointestinal distress [67]. Moreover, Almario et al. (2018) found heartburn/reflux commonly co-occurred with abdominal pain ((Population-Weighted; PW) 12.3%), bloat/gas (PW 10.2%), constipation (PW 9.2%), and diarrhea (PW 9.2%), leading to a complex presentation that may overlap with functional dyspepsia or other functional gastrointestinal disorders [32].

### 3.3. The Pharmacist’s Role in Heartburn Self-Diagnosis

While patients often self-diagnose heartburn, pharmacist confirmation and counseling may enhance the clinical accuracy of self-diagnosis and optimization of self-management. Pharmacist counseling for an individual experiencing heartburn should include reviewing the patient’s medical and medication use history, assessing symptom frequency, risk factors and severity, and distinguishing heartburn from other conditions.

To differentiate between heartburn and GERD or other more serious gastrointestinal conditions, pharmacists may consider referring patients to healthcare providers who may recommend additional clinical diagnostic testing, such as upper GI endoscopy, esophageal manometry, barium swallow studies, pH monitoring, and impedance testing. Such tests are particularly advised for cases unresponsive to OTC treatments, those presenting red flag symptoms (detailed in Table 3), or in complicated or recurrent scenarios [68]. The frequency of symptoms is essential in diagnosing heartburn, as it aids in categorizing the condition as episodic or frequent. Episodic heartburn typically, occurr sporadically (e.g., fewer than two occurrences per week), while frequent heartburn is defined by two or more heartburn episodes per week.

Pharmacists play a pivotal role in supporting patients by employing structured symptom assessments. These include asking targeted questions addressing symptom frequency, nature, and severity. For instance, the University of Saskatchewan has developed an assessment checklist, guided by recommendations from the ACG and the WGO, to help pharmacists evaluate reflux symptoms effectively [69]. These questions, detailed in Table 3, enable pharmacists to confirm self-diagnoses and devise suitable treatment plans and have been developed based upon data interpreted from previously published clinical studies and guidelines [1,70].

Upon confirming a self-diagnosis, pharmacists should guide patients in adopting effective self-management strategies for heartburn. These strategies include the use of OTC medications and lifestyle modifications, both of which can significantly enhance the patient’s quality of life.

**Table 3:** Guided Questions for Accurate Heartburn Diagnosis and Referral [1,70].

Questions
What is the age of the patient?
What is the gender of the patient?
What symptom(s) is the patient experiencing? − An uncomfortable feeling behind the breastbone moving upward from the stomach − A burning sensation in the back of throat − A bitter or acidic taste in mouth
How frequently do the symptoms occur? − One or two times per week − One or two times in two weeks − One or two times in a month
When does the patient experience heartburn? − After a hectic day at work − After or during business travel − After week of long hours at work − After eating a heavy, high fat meal − After consuming acidic foods or alcoholic beverages − After smoking − After lying down to sleep or during sleep
Does the patient experience heartburn after lying down for 1 to 3 h after eating? − Always − Frequently − Sometimes − Rarely − Never
Has the patient previously tried lifestyle changes or medications? How did they affect patient’s symptoms?
Referral criteria for further medical evaluation 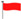 Pharmacists should refer patients for further medical evaluation, in lieu of self-care, if any of the following red flag symptoms or clinical considerations are present: − Patients who report experiencing frequent symptoms, which may be worsening over time − Patients experiencing any unintentional weight loss, difficulties in or painful swallowing, recurrent cough, hoarseness/changes in voice, blood in stools or vomiting − Patients with a family history of gastric and/or esophageal cancer − Patients who are pregnant or breastfeeding − Children

### 3.4. Heartburn Treatment Options

Effective heartburn treatment begins with a comprehensive assessment of the patient’s symptoms. This evaluation is crucial in determining whether self-treatment is appropriate or if the patient requires referral to a healthcare provider, especially when red flag symptoms (listed in Table 3) are present.

For cases suitable for self-treatment, pharmacists should recommend lifestyle and dietary modifications as foundational steps, ensuring these changes are maintained throughout the course of treatment [36]. However, when OTC medications are indicated, their selection should be guided by factors such as symptom frequency, duration, and severity, as well as considerations like cost, potential drug interactions, adverse effects, and the patient’s preference.

**Figure 2:**
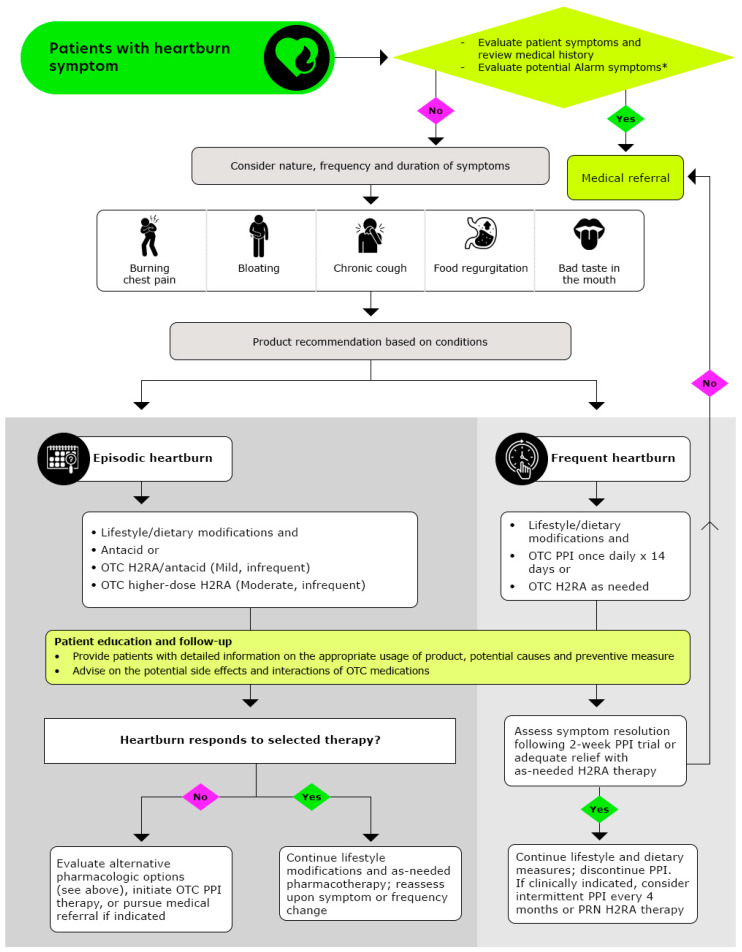
Algorithmic Approach for Pharmacists to Optimize Heartburn Treatment (Reproduced with permission from American Pharmacists Association, Handbook of Nonprescription Drugs, 21st edition heartburn chapter from ref: [71]); OTC: Over-the counter, H2RAs: Histamine-2-receptor antagonists, PPIs: Proton pump inhibitors; * Alarm symptoms: Patients who report experiencing frequent symptoms, which may be worsening over time, patients experiencing any unintentional weight loss, difficulties in or painful swallowing, re-current cough, hoarseness/changes in voice, blood in stools or vomiting, patients with a family history of gastric and/or esophageal cancer, patients who are pregnant or breastfeeding, patients with chest pain unrelieved by antacids or position, especially when accompanied by symptoms of sweating or breathlessness, warrants immediate evaluation for possible cardiac origin.

Pharmacists play a pivotal role in ensuring the safe and effective use of OTC medications by monitoring dosage and treatment duration, thereby enhancing patient safety and health outcomes. The recommended stepwise approach to managing heartburn, as followed by pharmacists, is outlined in Figure 2 (Reproduced with permission from American Pharmacists Association, Handbook of Nonprescription Drugs, 21st edition heartburn chapter) [71].

#### 3.4.1. Dietary and Lifestyle Modifications

The LES plays a pivotal role in protecting against reflux, and its relaxation can significantly influence the development of heartburn. Lifestyle interventions are essential in mitigating acid reflux and its associated symptoms by addressing factors that impact LES function. Two recent systematic reviews have reinforced the link between dietary factors—a key aspect of lifestyle modifications and reflux-like symptoms [53,72].

In 2008, the AGA Institute Council, along with the AGA Institute Clinical Practice and Quality Management Committee, issued recommendations for managing GERD symptoms, including heartburn. These recommendations emphasized specific lifestyle modifications, categorized into three distinct groups [73]:

(a) Avoidance of foods that may precipitate acid reflux

Certain foods like chocolate and peppermint, can relax the LES, increasing the likelihood of acid reflux. These foods should be avoided, especially after dinner, by individuals prone to heartburn [53]. Fatty foods, which delay gastric emptying and prolong stomach fullness, also elevate the risk of acid reflux. Similarly, consuming large meals increases intragastric pressure, further contributing to acid reflux [74]. Alcohol, known to lower LES pressure, also increases reflux risk. A systematic review and meta-analysis have confirmed the association between alcohol consumption and heartburn [75]. Therefore, limiting or eliminating fatty foods, large meals, or alcohol consumption can significantly reduce the occurrence of heartburn [76,77].

(b) Avoidance of acidic foods that irritate the esophagus

Acidic foods, such as citrus juices, carbonated beverages, and tomato-based products, can irritate the esophageal lining and exacerbate heartburn symptoms. Spicy foods, particularly those containing capsaicin, should also be avoided as they can increase gastric acid production and delay gastric emptying, thereby intensifying symptoms [46]. A recent cross-sectional study involving 280 GERD patients confirmed the association between GERD symptoms, such as heartburn, and the consumption of tomato-based products, citrus fruits/juices, and carbonated beverages [78].

(c) Actions to reduce esophageal acid exposure

Smoking has been shown to lower LES pressure, thereby increasing the risk of acid reflux. Therefore, smoking cessation is a key preventive strategy for heartburn. Central obesity, which elevates intra-abdominal pressure, particularly during activities like bending over or wearing tight clothing, can exacerbate reflux symptoms. Weight loss and opting for loose-fitting clothes are often the first-line recommendations to ease reflux or heartburn symptoms [3]. Elevating the head of the bed by 6 to 8 inches can also reduce reflux risk by utilizing gravity to keep gastric acid in the stomach. In the same way, avoiding recumbency for 2–3 h after meals prevents acid from moving into the esophagus.

According to an evidence-based consensus published in 2024, it is strongly recommended to refrain from the consumption of citrus fruits, tomatoes, heavily spiced dishes, fatty foods, fried items, chocolate, substantial meals, smoking, and alcoholic drinks for heartburn management. Furthermore, there is a moderate recommendation to limit the intake of coffee, carbonated drinks, and to adopt a low-fat diet [79].

(d) Medication review for drugs contributing to heartburn symptoms

When evaluating a patient’s symptoms and medication history, it is crucial for pharmacists to examine both previous and current medication usage as patients may have comorbid conditions that can lead to exacerbation of symptoms or suboptimal treatment response.

In recent years, there has been a significant increase in the prevalence of T2DM, which has been closely associated with a heightened risk of cardiovascular complications such as myocardial infarction and stroke [79]. This growing health burden has prompted extensive research and the development of novel therapeutic strategies. Among these, GLP-1RAs have emerged as promising agents due to their dual role in lowering glycated hemoglobin (HbA1c) levels and promoting weight reduction. Additionally, clinical trials have demonstrated their cardioprotective benefits in individuals with T2DM [80].

Despite these advantages, GLP-1RAs are frequently associated with gastrointestinal adverse effects, including dyspepsia or heartburn, likely due to their impact on gastric motility. Moreover, several other pharmacological agents have also been implicated in the onset or exacerbation of gastroesophageal reflux symptoms as previously discussed. The mechanisms by which medications can contribute to heartburn vary, ranging from relaxation of the LES to direct mucosal irritation [81,82].

The occurrence of such gastrointestinal side effects poses a significant challenge in clinical management, potentially compromising medication adherence and overall therapeutic outcomes. As a result, it is essential to implement strategies aimed at minimizing these adverse effects. Therefore, pharmacists should perform a thorough medication review to identify potential medication-related contributors to heartburn symptoms in order to provide appropriate advice or referrals for patients experiencing possible drug-induced symptoms [14,42,83].

In such cases, relying solely on lifestyle modifications is often insufficient for effective management. OTC medications play a pivotal role in these scenarios, offering symptomatic relief and complementing lifestyle changes in heartburn management.

#### 3.4.2. OTC Medications

Acid suppression is essential for managing heartburn and reflux symptoms. The WGO recommends antacids, H2RAs, and PPIs as suitable OTC options for infrequent, mild to moderate heartburn symptoms [6,31].

(a) Antacids

Antacids partially neutralize gastric acid and inhibit the enzyme pepsin, offering relief by neutralizing acid in the esophagus, not the stomach [84,85]. Common active ingredients include sodium bicarbonate, calcium carbonate, aluminum hydroxide, or magnesium hydroxide/carbonate salts. Antacids provide quick relief within 1 to 5 min with a duration of action lasting approximately 20 to 60 min, making them suitable for occasional heartburn [71,86]. Antacids are compared based on their acid-neutralizing capacity (ANC), defined as the amount of hydrochloric acid (HCl) (in milliequivalents, mEq) required to maintain 1 mL of an antacid suspension at pH 3 for 2 h in vitro [4].

Antacids are available in multiple dosage forms, including chewable tablets, liquids, gel caps, powders, and effervescent granules with various combinations of magnesium and aluminum salts [87]. Dosage form influences the onset and efficacy of symptom relief. For example, chewable antacids outperform liquid formulations in relieving heartburn and neutralizing esophageal acid [4,88,89].

Gel-cap antacids offer limited heartburn relief beyond the water consumed with them, as they do not neutralize esophageal acid. In contrast, chewable antacids, even at smaller doses, may provide better esophageal pH control and symptom relief than larger doses of gel-cap formulations [3,85]. Antacids can be taken multiple times a day, as indicated on product labels. Antacids are effective for infrequent heartburn and can serve as a backup for breakthrough episodes;however, they are not ideal for treating frequent heartburn. Subjects with persistent heartburn and warning symptoms of complicated reflux disease, such as regurgitation, dysphagia, odynophagia, wheezing, or persistent symptoms despite 4 to 6 weeks of lifestyle modifications and OTC medications use had significant erosive esophagitis (47%) or other complications of reflux disease [6,90]. Safety considerations associated with antacids are detailed in Table 4.

The 2013 WGO guidelines [31] recommend alginate-containing agents for heartburn management. However, 2022 ACG Clinical Guideline for GERD [1], mentions that alginic acid preparations showed potential efficacy in the United Kingdom, but alginate content of preparations sold in other countries is variable [1].

(b) Histamine-2-Receptor Antagonists (H2RAs)

The H2RAs reduce gastric acid production by competitively and reversibly binding to histamine-2 receptors on gastric parietal cells. They quickly elevate gastric pH within 30 min of administration, with effects lasting up to 10 h [31]. A single dose effectively relieves or prevents heartburn symptoms, though repeated use can lead to tolerance, and thus suboptimal treatment for frequent heartburn [3] H2RAs are most effective when taken before activities known to trigger heartburn, like heavy meals or late-night eating [91].

The FDA has approved OTC use of cimetidine and famotidine for heartburn treatment [92,93]. A Cochrane review on short-term treatment for GERD symptoms reported that H2RAs significantly improve heartburn remission compared to placebo, with a risk ratio of 0.77 (two trials; 95% CI, 0.60–0.99) [94]. They are generally well-tolerated, with less than 5% of users experiencing adverse effects such as confusion, headache, vertigo, and rare cases of thrombocytopenia [95]. Rare adverse effects, unrelated to dose or duration include hepatotoxicity, skin rash, bone marrow suppression, and pancreatitis. High doses of cimetidine have been associated with gynecomastia and impotence, both of which typically resolve upon discontinuation of therapy [96]. While vitamin B12 deficiency attributed to H2RAs has been described, the risk is greater with chronic high-dose PPI therapy [97]. Additionally, H2RA may rarely prolong the QTc interval, especially in those with renal impairment or when taken with concomitant QTc-prolonging meds. Clinicians should review cardiac history and medication profiles before use [98].

(c) Proton Pump Inhibitors (PPIs)

The PPIs irreversibly bind to active proton pumps on gastric parietal cells, effectively suppressing acid production [99]. In the use of the OTC PPIs for managing heartburn, these medications should be administered once daily, preferably in the morning and 30–60 min before the first meal, as administration after a prolonged fast may optimize efficacy [99,100]. PPIs attain peak serum levels approximately 12 h post-dose, with a half-life of 1 to 2 h. However, their biological half-life exceeds 24 h, as gastric parietal cells must synthesize new proton pumps to restore baseline acid secretion. The common cause of PPI failure is improper dosing, such as taking the medication without food (e.g., at bedtime). PPIs exhibit a dose-dependent effect on controlling gastric pH [101] and use may result in complete resolution of frequent heartburn symptoms [102,103,104]. As per the 2013 ACG treatment guidelines, the minimum effective PPI dose should be used for heartburn management [105].

PPIs are more effective than H2RAs in suppressing gastric acid and treating frequent heartburn [102]. Omeprazole’s acid suppression improved gradually over several days, eventually reaching a steady state, while famotidine’s efficacy declined with repeated dosing [106].

PPIs have also been evaluated in comparative studies to determine their relative efficacy. For instance, omeprazole (20.6 mg) was found to maintain gastric pH > 4 for a greater percentage of time than lansoprazole (15 mg) (46% vs. 37%, respectively) [101]. Another clinical trial compared the efficacy of omeprazole 10 mg or 20 mg administered daily for two weeks in patients with frequent heartburn. At the end of week two, 55% of patients taking omeprazole 20 mg were heartburn-free, compared to 40% on omeprazole 10 mg, demonstrating superior efficacy of the higher dose [102]. A larger trial involving 2645 patients reported no significant differences in achieving complete heartburn resolution between omeprazole 20 mg and esomeprazole, either 20 mg or 40 mg [107]. This highlights that while individual PPIs may vary in certain pharmacodynamic parameters, their overall clinical efficacy in resolving heartburn remains comparable in many cases.

PPIs are rapidly metabolized in the liver, primarily by CYP2C19 and CYP3A4 enzymes. Genetic differences in these enzymes classify individuals as either poor or extensive metabolizers of PPIs, affecting therapeutic response. Therefore, PPIs need extra monitoring in patients with liver impairment [108,109].

Common side effects associated with PPIs include headache, diarrhea, constipation, and abdominal pain. However, none of these adverse effects have been reported to occur more frequently than with placebo and are often resolved by switching to another PPI or by lowering the dose [73]. Prolonged PPI use may also affect electrolyte balance and contribute to QT interval changes, withsome data suggesting varying risk among the different PPI agents. Monitoring electrolytes and reviewing concurrent medications is advisable, especially in high-risk patients [110].

Long-term PPI use has raised concern regarding hypergastrinemia and associated carcinogenesis [95]. However, over 20 years of clinical use, there is no conclusive evidence linking PPIs to gastric carcinoid tumors, gastric adenocarcinoma, or colorectal carcinoma Although long-term high-dose PPI therapy has also been associated with an increased risk of hip fractures, this risk appears to be independent of significant bone mineral density loss, indicating that factors such as impaired calcium absorption, altered bone remodeling, or deterioration in bone microarchitecture may be more influential [111]. Furthermore, long-term high-dose PPI therapy has been associated with an increased risk of *Clostridioides difficile* colitis, vitamin B12 deficiency, and, in rare cases, acute interstitial nephritis [97,112,113].

**Table 4:** OTC medications available for the treatment of heartburn in U.S. ^#^ [114,115].

	Antacids				Histamine-2-Receptor Antagonists (H2RAs)		Proton Pump Inhibitors (PPIs)		
	Calcium salts	Sodium salts	Magnesium salts	Aluminum salts	Cimetidine	Famotidine	Omeprazole	Esomeprazole	Lansoprazole
Maximum daily nonprescription dosage	160 mEq	200 mEq (≤60 years old) and 100 mEq (>60 years or older)	50 mEq	NA	200–400 mg	40 mg	20 mg	20 mg	15 mg
Frequency	Varies based on product and formulation; used not more than 14 days for self-management of heartburn symptoms				Once a day 30–60 min before the first meal; used for no longer than 14 days for self-management of heartburn symptoms				
Side effects	Constipation, flatulence, systemic alkalosis, hypercalcemia, renal stones	Flatulence, gastric distension, systemic alkalosis and sodium overload with prolonged use	Diarrhea, hypermagnesemia, renal stones	Constipation, intestinal obstruction, hypophosphatemia	Diarrhea headache, hormonal imbalance	Fever, asthenia, fatigue, palpitations, elevated liver enzymes	Headache, abdominal pain, nausea, diarrhea, vomiting, and flatulence	Headache, diarrhea, nausea, flatulence, abdominal pain, constipation, and dry mouth	Diarrhea, abdominal pain, nausea, and constipation
Drug interaction	Reduced absorption of tetracyclines due to chelation with polyvalent cations (Ca^2+^, Mg^2+^, Al^3+^) Decreased bioavailability of fluoroquinolones (e.g., ciprofloxacin) by insoluble complex formation Lower serum concentration of bisphosphonates (e.g., alendronate): due to impaired absorption Poor dissolution of ketoconazole in elevated gastric pH thereby reduced efficacy Decreased absorption of HIV Protease Inhibitors (e.g., atazanavir) due to increased gastric pH Reduced bioavailability of dasatinib and imatinib due to pH-dependent solubility Chelation with antacid cations leads to decreased systemic exposure of eltrombopag Altered permeability and absorption depending of sulfonylureas based on antacid type and gastric pH				Elevated plasma level of phenytoin and risk of toxicity Increased serum concentration of theophylline, with potential CNS and cardiac side effects Prolonged sedation caused by benzodiazepines (e.g., diazepam) due to reduced metabolism Cimetidine reduced the renal clearance of several medications (e.g., procainamide, quinidine, propranolol, verapamil, others) Famotidine may contribute to QTc prolongation particularly when used with other QTc prolongation drugs, or in those with poor kidney function		Reduced efficacy of clopidogrel due to CYP2C19 inhibition Poor absorption of antiretrovirals due to increase in gastric pH Lower bioavailability of antifungals due to acidic stomach Increased serum concentration of antiepileptics Impaired absorption of iron supplements Reduced clearance and increase associated toxicity risk due to methotrexate Lansoprazole, has been associated with QTc interval prolongation in ICU patients, warranting caution with other QTc-prolonging drugs.		
Warnings	Ask a healthcare professional before use if you have kidney stone or on a calcium-restricted diet				Do not use if allergic to cimetidine or other acid reducers	Do not use if allergic to famotidine or other acid reducers	-*Clostridioides difficile* associated diarrhea -Acute tubular nephritis and risk of fractures with long term therapy -Known hypersensitivity to substituted benzimidazoles or any component of the formulation		
Contraindications *									
Renal Impairment	No	Yes	Yes	Yes	No	Yes	No	No	No
Hepatic Impairment	No	Yes	No	No	No	No	Yes	Yes	Yes
Other considerations for use **	-Patients with hypercalcemia, hypercalciuria, nephrocalcinosis, and nephrolithiasis -Patients on a low-phosphate diet	-Patients on a sodium- restricted diet, e.g., those with hypertension or congestive heart failure	-Patients with severe diarrhea -Patients with neuromuscular disease such as myasthenia gravis	-Patients with constipation	-Patient with pain in swallowing food, vomiting with blood, or black stools or chest pain or lightheadedness, sweating or dizziness				

NA: Not available; mEq: Milliequivalents. * For a comprehensive and systematic account of contraindications, it is suggested to follow the official prescribing information. ** Not an exhaustive list. ^#^ Source: Package labels [111,112].

### 3.5. The Role of the Pharmacist in Heartburn Management

Patients suffering from heartburn or acid reflux frequently turn to pharmacists for assistance, particularly when their daily lives are significantly disrupted. Pharmacists are uniquely positioned to guide these patients by confirming diagnoses, referring individuals with alarming symptoms for further medical evaluation, and educating them on self-care principles, including the appropriate selection and use of OTC heartburn medications [116]. With the increasing availability of medications transitioning from prescription-only to OTC status, the pharmacist’s role in heartburn management has expanded. Patients’ choices regarding self-care and self-medication have a profound impact on their health outcomes. When pharmacists actively engage with patients to encourage adherence and proper use of medications, better health outcomes are achieved. The increasing availability of OTC pharmacologic options for short-term heartburn symptom management places greater responsibility on pharmacists to guide patients towards appropriate choices. During patient consultations, pharmacists should practice effective listening, ideally in a setting conducive to open communication and address patients’ questions regarding the safe and effective use of OTC medications. Communication empowers patients and may motivate them to actively participate in decisions about their healthcare and self-management of heartburn symptoms. To fulfill this role effectively, pharmacists must stay informed about the latest available products and the evidence supporting their use in OTC settings.

The key responsibilities of pharmacists in managing heartburn include the following [117].

#### 3.5.1. Information Collection and Symptom Assessment

Pharmacists should gather and evaluate details about the patient’s primary complaint and general health to determine the most appropriate course of action, including triage when necessary. By taking a detailed history and assessing symptoms, pharmacists can help patients differentiate between heartburn and other conditions with similar presentations, such as GERD or non-erosive reflux disease. Patients exhibiting “red flag” or alarm symptoms, such as persistent pain, dysphagia, or unexplained weight loss, should be referred to their primary healthcare provider for further evaluation. Notably, studies show that 59.5% of patients misinterpreted myocardial infarction (MI) pain as heartburn during their first episode, though this dropped to 3.3% in subsequent attacks [118]. This underscores the need for accurate symptom recognition, particularly at initial presentation. Whereas heartburn typically presents as a burning sensation in the chest that improves with antacids or positional change, MI pain is often described as sudden, pressure-like discomfort that may radiate to the arms, jaw, or neck and is unresponsive to such measures. Importantly, chest pain unrelieved by antacids or accompanied by systemic symptoms (e.g., chest tightness, sweating, or shortness of breath) should prompt immediate medical evaluation to rule out cardiac causes and prevent potentially life-threatening delays in treatment.

#### 3.5.2. Lifestyle Advice

Pharmacists may advise patients on lifestyle changes to manage heartburn, including dietary modifications, weight loss, and avoidance of trigger foods and beverages.

#### 3.5.3. Guidance on OTC Medication Selection and Use

Pharmacists should guide patients in selecting the most suitable OTC heartburn medications based on evaluation of symptoms and needs. It may be helpful to explain the differences in medication mechanisms and their appropriate uses (Table 4). Multiple studies underscore the pharmacist’s expanded role as a clinical advisor, educator, and safety monitor in OTC medication management. A recent review emphasizes their responsibility in ensuring effective and responsible self-care, particularly with OTC PPIs.

Nakhla et al. 2024 [119] reported that pharmacists in Ontario and Québec routinely evaluate heartburn severity and recommend appropriate OTC options. The study also found that pharmacists frequently advised switching therapies or referring patients after seven days of persistent symptoms, highlighting their role in monitoring treatment and ensuring safety. Survey data further revealed pharmacists’ confidence in managing heartburn cases, reinforcing their status as trusted advisors in OTC therapy selection and patient education.

Pharmacists should additionally stress the importance of adherence to medication regimens, as non-adherence can lead to health risks and increase healthcare costs.

#### 3.5.4. Safety Monitoring

Pharmacists should educate patients about potential side effects and drug interactions associated with OTC heartburn medications, emphasizing the importance of reading product labels carefully and advising patients to seek further medical attention if symptoms persist or worsen (e.g., persistent heartburn, difficulty swallowing, or unexplained weight loss).

Safety considerations associated with OTC heartburn medications, including potential interactions and warnings, are detailed in Table 4.

### 3.6. Special Groups

Heartburn management requires tailored approaches for specific patient groups due to physiological differences, safety concerns, and comorbidities. Evidence-based guidelines and expert consensus provide structured therapeutic algorithms for pregnant women, children, older adults, and individuals with chronic diseases.

Physiological changes during pregnancy can intensify heartburn symptoms, often requiring tailored adjustments to lifestyle and dietary habits, as well as the use of non-pharmacological and pharmacological interventions. This necessitates careful modification of standard drug therapy approaches and underscores the importance of identifying safer therapeutic alternatives for pregnant women [120]. Self-care for this population should begin with lifestyle changes and antacids, favoring calcium or magnesium formulations while avoiding chronic aluminum exposure. If symptoms persist, famotidine is the preferred H_2_RA due to its favorable safety profile. Data on the use of PPIs during pregnancy are limited; PPIs should be reserved for refractory cases, used at the lowest effective doses and discontinued postpartum [121,122]. Importantly, clinical guidance must reinforce OTC labeling, which advises pregnant and breastfeeding women to consult a healthcare professional before use. This precaution should be clearly emphasized in both provider recommendations and patient education to support informed decision-making.

Similarly, in children and adolescents, the North American Society for Pediatric Gastroenterology, Hepatology, and Nutrition (NASPGHAN) and the European Society for Pediatric Gastroenterology, Hepatology, and Nutrition (ESPGHAN) algorithms emphasize non-pharmacologic strategies like thickened feed. For children older than age 2 experiencing episodic heartburn symptoms, in addition to lifestyle intervention previously mentioned, a calcium-carbonate containing antacid product formulated for children may be considered; persisting symptoms warrant referral to their pediatrician or healthcare provider. For children experiencing heartburn, OTC product labeling must be strictly followed. For example, OTC H2RAs are not indicated for children younger than age 12 and PPIs are not recommended for use in individuals under 18 years of age. Emphasizing age-specific guidance helps ensure safe and appropriate use of OTC medications for the pediatric patient experiencing heartburn symptoms [123,124].

In older adults, medication use and underlying medical conditions may represent factors contributing to heartburn symptoms. For these patients, careful consideration of concomitant medication use and underlying renal function are paramount in evaluating appropriateness for self-care. For intermittent symptoms, antacids or H2RAs could be considered, depending on kidney function, while short-term use of the PPIs remain first-line for frequent heartburn symptoms; persisting or red flag symptoms warrant further medication evaluation. Chronic, persistent use of H2RAs and PPIs additionally warrant periodic re-evaluation or deprescribing to limit adverse outcomes. Among the PPIs, pantoprazole is significantly effective both for acute and long-term treatment with excellent control of relapse and symptoms. It is generally well tolerated, even with extended use, and its safety profile is considered optimal [125]. Nonetheless, pharmacists’ should adhere to OTC product labeling and the 2023 Beers Criteria guidance to avoid inappropriate long-term use and reduce the risk of potential complications such as *Clostridioides difficile* infection, bone fractures, and possible cognitive decline [126]

## 4. Conclusions

Pharmacists are positioned to guide patient self-care for heartburn symptom management, including guiding the selection of optimal OTC treatment, by confirming the diagnosis, referring patients with alarm symptoms to physicians, and educating patients on the proper use of these medications. Given the high prevalence of heartburn and tendency for patients to self-diagnose and self-medicate, the wide range of available OTC options can lead to confusion and inappropriate use, potentially leading to medication errors. Pharmacists play a pivotal role in heartburn management by guiding self-care decisions through expert evaluation, education, and tailored treatment recommendations. With the expanding scope of pharmacy practice including medication therapy management and pharmacovigilance there is a growing need for concise, clinically relevant resources. This updated review is intended to provide a comprehensive resource on heartburn epidemiology, risk factors, pathophysiology, and treatment strategies, while emphasizing the pharmacist’s role in patient education and early detection of serious conditions.

By bridging the gap between patients and the healthcare system, pharmacists enhance patient care through education and personalized support leading to better health outcomes and improved quality of care. Future efforts should focus on further integrating pharmacists into patient care teams and expanding the role of the community and ambulatory care pharmacist in public health education to maximize their impact on heartburn management.

## 5. Limitations and Future Directions

This review synthesizes recent literature to highlight the pathophysiology, risk factors, and management strategies for heartburn, with a particular focus on the pharmacist’s evolving role. The selection of studies may be subject to publication and language bias. We restricted our search to English-language, peer-reviewed sources indexed in PubMed and Embase up to 1 March 2025 and did not carry out duplicate independent screening or a formal risk-of-bias appraisal. Grey literature, conference abstracts, and non-English guidelines may therefore be under-represented. Heterogeneity in study design and outcome definitions precluded a quantitative meta-analysis; consequently, recommendations are based on qualitative synthesis and should be interpreted with caution.

Despite these limitations, this review offers a valuable framework for pharmacists by integrating clinical insights and evidence-based strategies for guiding OTC heartburn management. However, much of the existing literature is centered on short-term outcomes such as symptom relief or acid suppression with limited focus on long-term patient adherence, esophageal health, or healthcare utilization.

Future research should aim to (1) evaluate the long-term effectiveness and safety of OTC heartburn interventions in diverse populations; (2) examine the real-world impact of pharmacist-led self-care counseling on clinical outcomes and healthcare costs; (3) explore innovative models for integrating pharmacists into multidisciplinary care teams for gastrointestinal health. Additionally, structured, high-quality systematic reviews and longitudinal studies are warranted to inform evidence-based practice and support guideline development.

## Data Availability

No new data was created or analyzed in this study. Data sharing is not applicable to this article.

## Supplementary Materials

The following supporting information can be downloaded at: https://www.mdpi.com/article/10.3390/pharmacy13050124/s1. Table S1: Scale for the Assessment of Narrative Review Articles—SANRA.
