# Supplementary material for: Empowering Pharmacists in Heartburn Management: Practical Insights for OTC Treatment and Self-Care
Source: Pharmacy (Basel). 2025 Sep 2;13(5):124. doi: 10.3390/pharmacy13050124 (PMC12452338; doi:10.3390/pharmacy13050124)
Supplement: Supplementary file 1 [file pharmacy-13-00124-s001.zip › pharmacy-3711590-supplementary.pdf]

Supplementary Materials

Supplementary Table S1: Scale for the Assessment of Narrative Review Articles – SANRA

Rating sheet

No.	Assessment item	0	1	2
1	Justification of the article's importance for the readership	The importance is not justified.	The importance is alluded to, but not explicitly justified.	**The importance is explicitly justified.** (*introduction section, 3rd paragraph, line no 68-87, 5th paragraph, line no 107-117))*
2	Statement of concrete aims or formulation of questions	No aims or questions are formulated.	Aims are formulated generally but not concretely or in terms of clear questions.	**One or more concrete aims or questions are formulated.** (*introduction section, last paragraph, line no-118-122))*
3	Description of the literature search	The search strategy is not presented.	The literature search is described briefly.	**The literature search is described in detail, including search terms and inclusion criteria.** (*material and methods section, paragraph 2.2)*
4	Referencing	Key statements are not supported by references.	Referencing of key statements is inconsistent.	**Key statements are supported by references.** (*reference list)*
5	Scientific reasoning (e.g., incorporation of appropriate evidence such as RCTs)	The article's point is not based on appropriate arguments.	Appropriate evidence is introduced selectively.	**Appropriate evidence is generally present.** *(results section)*
6	Appropriate presentation of data (e.g., absolute vs relative risk; effect sizes without CIs)	Data are presented inadequately.	*Data are often not presented in the most appropriate way.*	***Relevant outcome data are generally presented appropriately.*** *(Tables 1, 2, 3)*
